# CircDTL Functions as an Oncogene and Regulates Both Apoptosis and Ferroptosis in Non-small Cell Lung Cancer Cells

**DOI:** 10.3389/fgene.2021.743505

**Published:** 2021-09-21

**Authors:** Wang Shanshan, Ma Hongying, Fang Jingjing, Yu Yiming, Ren Yu, Yu Rui

**Affiliations:** ^1^The Affiliated Hospital of Medical School, Ningbo University, Ningbo, China; ^2^Department of Urologic Surgery, Ningbo Urology and Nephrology Hospital, Ningbo, China; ^3^Department of Biochemistry and Molecular Biology, Medical School of Ningbo University, Ningbo, China

**Keywords:** apoptosis, circDTL, ferroptosis, non-small cell lung cancer, miRNA

## Abstract

**Background:** Circular RNAs (circRNA) play an essential role in the tumorigenesis of non-small cell lung cancer (NSCLC). CircDTL is a novel identified circRNA with little information regarding its biological role. However, the role of circDTL in NSCLC has not been investigated yet.

**Method:** In this study, the levels of circDTL in tissues and cells were measured by RT-PCR. Cell viability was measured by the CCK-8 assay. Cell migration and invasion were evaluated using the wound healing assay and transwell assay, respectively. Cell death was measured by the cell death ELISA kit. The levels of Fe^2+^, ROS, MDA and GSH were measured using the commercial kits. The interactions between miR-1287-5p and circDTL/3′UTR GPX4 were verified by dual-luciferase activity assay. The effects of circDTL on tumor growth were evaluated *in vivo.*

**Results:** CircDTL was found to be upregulated and acted as an oncogene in NSCLC cells. Knockdown of circDTL promoted both apoptosis and ferroptosis of NSCLC cells. It was identified that circDTL exerts its oncogenic effects via the circDTL/miR-1287-5p/GPX4 axis and GPX4 inhibits both ferroptosis and apoptosis. Finally, this study showed that silencing of circDTL promoted the sensitivity of NSCLC cells to chemotherapeutic agents and inhibited the growth of tumors *in vivo*.

**Conclusion:** CircDTL acts as an oncogene and exerts its effects via the miR-1287-5p/GPX4 axis in NSCLC, providing a potential therapeutic target for NSCLC cancer therapy.

## Introduction

Lung cancer is the most prevalent diagnosed cancer and the leading cause of cancer-related death worldwide (Siegel et al., 2019). Non-small-cell lung carcinoma (NSCLC) accounts for nearly 85% of lung cancers and the prognosis of NSCLC is still dismal (Keith and Miller, 2013). Therefore, it is important to unveil the molecular mechanisms underlying the progression of NSCLC.

Circular RNA (circRNA) is a group of non-coding RNA featuring a closed-loop structure. Circular RNAs are more stable than linear RNAs due to their circular shape. CircRNAs have been shown to perform a variety of biological roles, including sponging miRNA, regulating RNA binding protein, and transcription in nuclear (Hsiao et al., 2017). Several studies have suggested that dysregulation of circRNAs is correlated to the development of NSCLC. CircDTL is a novel circRNA that has been reported to be dysregulated in medulloblastoma, but its role in NSCLC remains unknown, and further investigation is needed to understand its underlying mechanism (Lv et al., 2018).

MicroRNAs (miRNAs) are another form of small non-coding RNAs that regulate various biological processes such as differentiation, migration, metabolism, and programmed cell death (Hammond, 2015). Various miRNAs have been reported to participate in the tumorigenesis of cancers. miR-1287-5p has been found dysregulated and played essential roles in multiple cancers. For instance, miR-1287-5p inhibited the development of triple-negative breast cancer via inhibition of phosphoinositide-3-kinase CB (Schwarzenbacher et al., 2019). Another study found that miR-1287-5p could regulate the progression of cervical cancer via regulation of HOXA7 (Ji et al., 2020). However, there’s little knowledge about the role of miR-1287-5p in NSCLC.

Ferroptosis is a novel type of regulated cell death mediated by reactive oxygen species (ROS) and lipid peroxidation (Cao and Dixon, 2016). Glutathione peroxidase 4 (GPX4), a glutathione peroxidase, belongs to the GPX family and plays an essential role in the process of ferroptosis (Yang et al., 2014). GPX4 is able to convert lipid hydroperoxides to lipid alcohols. Therefore, inhibition of GPX4 can lead to the accumulation of lipid peroxides and thereby promote the ferroptosis. Various studies have shown that induction of ferroptosis via targeting GPX4 might be a promising strategy for killing cancer cells including the NSCLC (Hassannia et al., 2019). For example, blockage of GPX4 could overcome resistance to Lapatinib via promoting ferroptosis in NSCLC (Ni et al., 2021). Another study also found that ammonium ferric citrate induced ferroptosis via blockage of GPX4 in NSCLC cells (Wu et al., 2021). Interestingly, several researchers have found that non-coding RNAs also play essential roles in regulating ferroptosis (Wang et al., 2019; Lu et al., 2020). However, there’s still no investigation into the effects of circDTL on ferroptosis.

This study found that circDTL was upregulated and acted as an oncogene during the progression of NSCLC. Interestingly, silencing of circDTL induced ferroptosis of NSCLC cells and further investigation showed that circDTL regulated ferroptosis via the miR-1287-5p/GPX4 axis. These findings underlined the importance of circRNAs in the process of ferroptosis and introduced circDTL as a regulator of ferroptosis in NSCLC.

## Materials and Methods

### Clinical Specimens

A total of 84 pairs of NSCLC tissues and adjacent normal tissues were obtained from the Affiliated Hospital of Ningbo University. Before the operation, no treatment was given to any of the patients. This study was authorized by The Ethics Committee of Ningbo University and conducted following the Declaration of Helsinki. Before the study, all participants signed written informed consents.

### Cell Culture

Normal human lung epithelial cell line BEAS-2B and human NSCLC cell lines (H23, H522, PC9, and A549) were purchased from the Shanghai Bank of Cell Culture (Shanghai, China). All cells were cultured in RPMI1640 medium (Gibco, United States) supplemented with 10% fetal bovine serum (FBS, Gibco), 100 U/ml penicillin and 100 μg/ml streptomycin (Invitrogen, United States). At 37°C, the cells were kept in a humidity-controlled environment with 5% CO_2_.

### RNA Purification and RT-PCR

Total RNA was extracted using Trizol (Invitrogen, United States). RNA was reversely transcribed into cDNA using PrimeScript RT Reagent (Takara, China). The expression levels of circDTL and miRNAs were evaluated using the SYBR Premix Ex Taq (Takara, China). Internal controls were GAPDH and U6. The relative gene expression was calculated using the 2^–ΔΔ*Ct*^ method and the experiments were performed in triplicate.

### Cell Transfection

shRNAs against circDTL were sub-cloned into the GV248 (hU6- MCS-Ubiquitin-EGFP-IRES-puromycin) construct (Genechem, China). The full length of GPX4 was synthesized by GenePharm (China) and sub-cloned into the GV248 vector. The QuickChange site-directed mutagenesis kit (Agilent, United States) was used to create Mutant GRPX4. The plasmids were transfected into HEK-293 cells using the ViraPower Kit (Life Technologies), to generate the lentivirus. GenePharm supplied miR-1287-5p mimics/inhibitors and negative controls (NC-mimics/inhibitors), and transfection was performed using the Lipofectamine 2000 (Life Technologies) according to the manufacturer’s instruction.

### RNA Pull-Down Assay

The RNA pull-down assay was performed according to the protocol from GeneSeed (Guangzhou, China). Following formaldehyde fixation, the cells were sonicated. Then, the supernatant was incubated with the biotinylated circDTL or control probe (RioBio) and the magnetic streptavidin Dyna beads (Sigma). After total RNA extraction, qRT-PCR was used to assess the enrichment.

### Cell Viability Assay

The viability of the cells was determined according to a previously published article (Yu et al., 2016). The cells were plated into 96-well plates at a density of 1 × 10^4^ cells/well. After culturing for 24 h, cells were subjected to different treatments. 10 μl of CCK-8 solution was added to each well and incubated at 37°C for another 2 h. Then the absorbance was measured at 450 nm using a microplate reader (Biotek, United States).

### Cellular Death Assay

The cellular death was measured by the Cell Death Detection ELISA^p*lus*^ kit (Roche, Germany), following the manufacturer’s guide.

### Measurement of ROS, MDA, GSH, and Fe^2+^

The levels of ROS, MDA, GSH, and Fe^2+^ were measured using the Cellular ROS Assay Kit (Abcam, United States), Lipid Peroxidation (MDA) Assay Kit (Abcam, United States), GSH Assay Kit (Abcam, United States), and Iron Assay Kit (Abcam, United States), respectively.

### Sub-Cellular Fraction Assay

The location of RNAs was determined using the PARIS^TM^ kit (Invitrogen), according to the company’s manual. Cells were suspended in cytoplasm lysis buffer and centrifuged at 1,500 rpm for 5 min. The cytoplasmic supernatant was collected, and the pellet was re-suspended in nucleus lysis buffer at 4°C for 1 h after centrifugation at 1,500 rpm for 10 min. The RNAs derived from cytoplasmic and nuclear extracts were purified by TRIzol. The expression levels of GAPDH (cytoplasm control), U6 (nucleus control) and circDTL in the nucleus and cytoplasm were assayed by qRT-PCR.

### Dual-Luciferase Activity Assay

The wild-type (including miR-1287-5p binding sites) sequences of circDTL whole length or GPX4 3′UTR region were sub-cloned into the pmirGLO vector (Life Technologies). NSCLC cells were co-transfected with luciferase reporters along with miR-1287-5p mimics (wild type or mutants) or miR-NC using Lipofectamine 2000 according to the company’s protocol. The Dual-Luciferase Reporter Assay System (System) was used to adjust relative luciferase activity to firefly luciferase activity 48 h after transfection (Promega Madison, WI, United States).

### Cell Migration and Invasion Assay

The migration of cells was measured by the wound healing assay. After transfection for 24 h, cells were seeded into a 6-well plate at the density of 2 × 10^5^ cells/well. 24 The monolayer was scratched with a sterile 20 l pipette tip 24 h later. Cell migration was observed 24 h later. Invasion of cells was assayed using the Transwell assay. 1 × 10^5^ transfected cells were suspended in 200 μl of serum-free medium and seeded into the top chambers of Transwell (8 μm pore size, Corning, United States) coated with Matrigel (BD Bioscience). As an attractant, the bottom chamber was filled with the entire medium. After incubation for 24 h, non-invaded cells were gently removed and cells invaded were fixed with 4% paraformaldehyde (Sigma), stained with crystal violet solution (Beyotime) for 30 min, and visualized. All cells were counted in five microscopic areas selected at random.

### Western Blot Assay

The RIPA lysis buffer was used to lyse the cells (Beyotime). SDS-PAGE was used to separate 20 g of total protein, which was then transferred to the PVDF membrane. The membranes were blocked with skimmed milk for 1 h at room temperature, and then the membrane was incubated with primary antibody overnight at 4°C. After that, the membrane was washed three times with PBS and incubated with corresponding HRP-conjugated secondary antibody at room temperature for 1 h. ECL Prime Western Blotting Kit was used to view the results (Beyotime). Cellular Signaling Technology, United States (CST) provided all of the main and secondary antibodies.

### Animal Study

The Shanghai SLAC Animal Center (Shanghai, China) provided 4–6-week-old BALB/c male nude mice, which were kept according to the standards for the use and care of laboratory animals. A total of 1 × 10^7^ NSCLC cells infected with shRNA were injected subcutaneously into the left flank of nude mice (3 per group). Every 3 days, the tumor volume was measured. After 30 days, all naked mice were killed, and the tumor tissues were analyzed by western blotting. These experiments gained permission from the committee on the Ethics of Animal Experiments of Affiliated Hospital of Ningbo University. The animal experiments were conducted according to the U.K Animals Act 1986 and associated guidelines.

### Statistically Analysis

Statistical analyses were performed with SPSS12.0 (IBM, Chicago, IL, United States). Data are expressed as the mean ± SD. A one-way ANOVA analysis was used to determine the statistical difference between multiple groups. The statistical difference between the two groups was calculated using a *post hoc* test. *P*-value < 0.05 (two-tailed) was considered statistically significant.

## Results

### CircDTL Was Upregulated and Acted as an Oncogene in NSCLC

**FIGURE 1:**
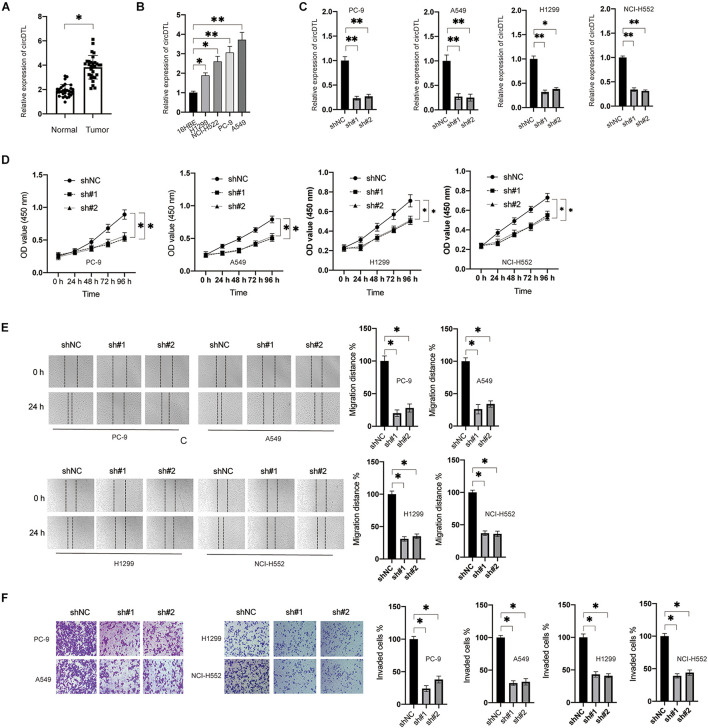
circDTL was upregulated and acted as an oncogene in NSCLC. **(A)** Levels of circDTL in 84 pairs of NSCLC and adjacent tissues. **(B)** Levels of circDTL in normal cell line BEAS-2B and NSCLC cell lines. **(C)** NSCLC cells were transfected with shNC or shRNA against circDTL (sh#1, sh#2) for 24 h, levels of circDTL were measured. **(D)** NSCLC cells were transfected as indicated and cell viability was measured at different time points. **(E)** NSCLC cells were transfected as indicated, cell migration was assayed. **(F)** Cell invasion was measured. Data were presented as mean ± SD. Experiments were performed at least three times. ^∗^*P* < 0.05; ^∗∗^*P* < 0.01.

The expression of circDTL was first assessed in 84 pairs of NSCLC tissues and surrounding normal tissues. The levels of circDTL were observed to be greater in NSCLC tissues than in normal tissues (Figure 1A). The levels of circDTL in NSCLC cells (H1299, NCI-H522, PC9, and A549) were also higher than human lung epithelial cell line BEAS-2B (Figure 1B). Two shRNAs targeting circDTL were utilized to knock down circDTL in NSCLC cells to study the biological role of circDTL in NSCLC (Figure 1C). Cell viability assays showed that downregulation of circDTL significantly inhibited the viabilities of NSCLC cells compared with the control groups (Figure 1D). Moreover, wound healing and Transwell assays showed that downregulation of circDTL repressed the migration and invasion of NSCLC cells (Figures 1E,F). These findings showed that circDTL might have a role in NSCLC as an oncogene.

### Inhibition of circDTL Induced Apoptosis and Ferroptosis in NSCLC Cells

**FIGURE 2:**
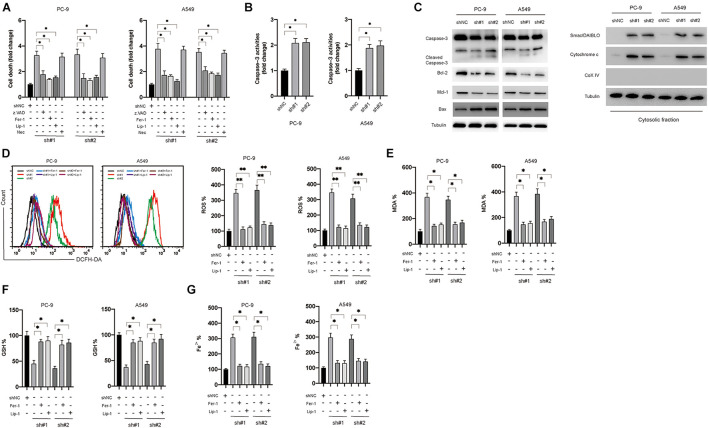
Silencing of circDTL induced apoptosis and ferroptosis of NSCLC cells. **(A)** NSCLC cells were transfected as indicated with or without different inhibitors for 24 h, cellular death was assayed. **(B)** NSCLC cells were transfected as indicated 24 h, caspase-3 activities were measured. **(C)** Apoptosis-related proteins were measured by western blots. **(D)** NSCLC cells were transfected as indicated and treated with or without ferroptosis inhibitors (Fer-1, Lip-1) for 24 h, ROS levels were measured. **(E)** MDA levels. **(F)** GSH levels. **(G)** Fe^2+^ levels. Data were presented as mean ± SD. Experiments were performed at least three times. ^∗^*P* < 0.05; ^∗∗^*P* < 0.01.

The effects of knockdown of circDTL on cell death of NSCLC cells were evaluated. Silencing of the circDTL gene resulted in greater cell death than control groups (Figure 2A). To determine the cell death type caused by the knockdown of circDTL, various specific inhibitors were applied. Apoptosis inhibitor (z.VAD.FMK, 10 μM) and ferroptosis inhibitors (Fer-1 10 μM, Lip-1 20 μM) but not necrosis inhibitor (Necrosulfonamide, NEC) significantly attenuated the cell death caused by knockdown of circDTL in NSCLC cells (Figure 2A). Caspase-3 activity assays and western blots confirmed that knockdown of circDTL triggered activation of caspase-3 in NSCLC cells (Figures 2B,C). Simultaneously, it was shown that silencing circDTL resulted in the downregulation of Bcl-2 and Mcl-1, as well as the overexpression of Bax (Figure 2C). Silencing of circDTL also led to the release of mitochondrial proteins Smac/DIABLO and cytochrome c into the cytosol (Figure 2C). Hence, knocking down circDTL triggered apoptosis in NSCLC cells via the intrinsic apoptotic mechanism. To confirm whether ferroptosis was also triggered by silencing of circDTL in NSCLC cells, the levels of ROS, MDA, GSH, and Fe^2+^ were assayed. It was found that knockdown of circDTL led to the upregulation of cellular ROS, which could be counteracted by Fer-1 or Lip-1 (Figure 2D). Silencing of circDTL also increased the levels of MDA, Fe^2+^ and decreased GSH levels, and those effects could be reversed by Fer-1 and Lip-1 (Figures 2E–G). These findings showed that inhibiting circDTL caused NSCLC cells to undergo both apoptosis and ferroptosis.

### circDTL Negatively Regulate the Expression of miR-1287-5p in NSCLC Cells

**FIGURE 3:**
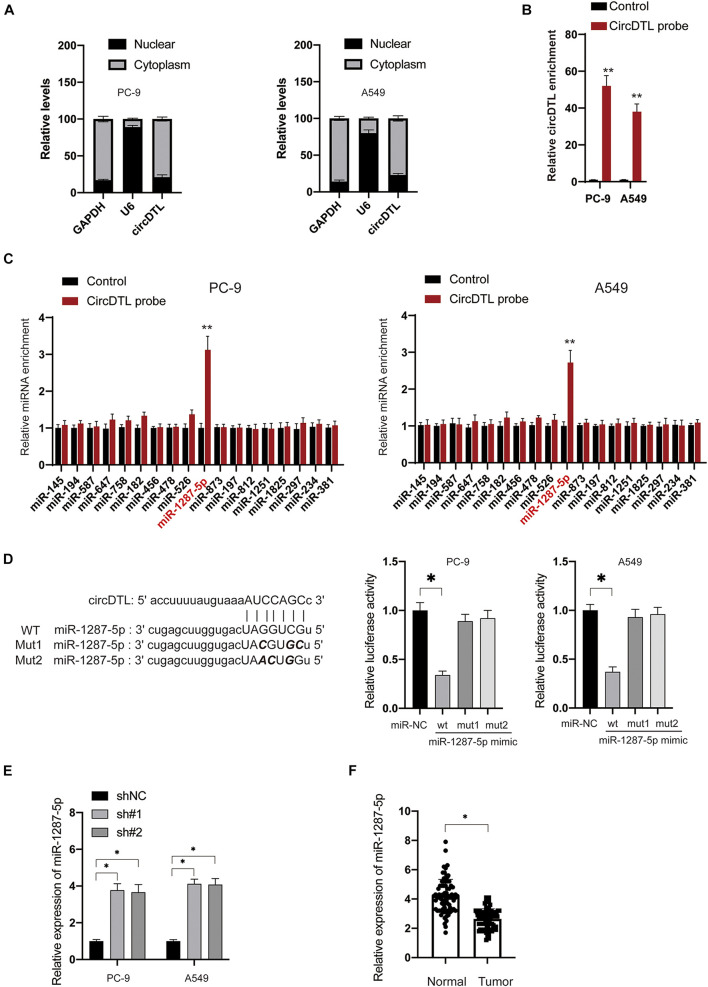
circDTL negatively regulates the expression of miR-1287-5p in NSCLC cells. **(A)** Cellular location of GAPDH, U6, and circDTL was assayed in NSCLC cells. **(B)** RNA pull-down assay was used for the detection of circDTL. **(C)** RNA pull-down was performed to examine different miRNAs that bind with circDTL. **(D)** Predictive binding sites between circDTL and miR-1287-5p (left), the interaction between miR-1287-5p and circDTL was evaluated by dual-luciferase reporter assay (right). **(E)** NSCLC cells were transfected with shNC or shRNAs against circDTL (sh#1, sh#2) for 24 h, levels of miR-1287-5p were measured. **(F)** The expression of miR-1287-5p in 84 pairs of NSCLC and adjacent normal tissues. Data were presented as mean ± SD. Experiments were performed at least three times. ^∗^*P* < 0.05; ^∗∗^*P* < 0.01.

Several studies have suggested that circDTL acts as a “sponge” to regulate the expression of miRNAs. A sub-cellular fractionation experiment was used to confirm this, and it revealed that circDTL is mostly found in the cytoplasm of NSCLC cells (Figure 3A). Then, using bioinformatical tools (StarBase3.0, TargetScan), potential miRNAs that can bind to circDTL were predicted (Supplementary File 1). RNA pull-down assays showed that specific probes against circDTL could enrich circDTLRNAs over 30-fold changes compared with control (Figure 3B). We chose those predicted miRNAs with an Ago CLIP-seq Data ≥ 5 and have been previously reported involved in the tumorigenesis of NSCLC to further analysis. Among them, miR-1287-5p was found to be significantly elevated in the circDTL probe group, whereas other miRNAs showed minimal change (Figure 3C). To further test that miR-1287-5p can bind with circDTL, two mutant miR-1287-5p mimics (Mut1, Mut2) containing mutations within the binding site for circDTL and a wild-type (WT) miR-1287-5p mimics were transfected into NSCLC cells (Figure 3D, left). It was found that only the WT miR-1287-5p mimic but not the mutants, inhibited circDTL luciferase reporter activity (Figure 3D, right). To further, elucidate the correlation between miR-1287-5p and circDTL, the expression of miR-1287-5p after knockdown of circDTL was examined. The inhibition of circDTL led to the upregulation of miR-1287-5p (Figure 3E). In addition, the expression of miR-1287-5p in normal tissues was much higher than NSCLC tissues (Figure 3F). These data suggested that circDTL acts as a “sponge” to negatively regulate the expression of miR-1287-5p.

### miR-1287-5p Targets GPX4 Which Regulates the Ferroptosis and Apoptosis of NSCLC Cells

**FIGURE 4:**
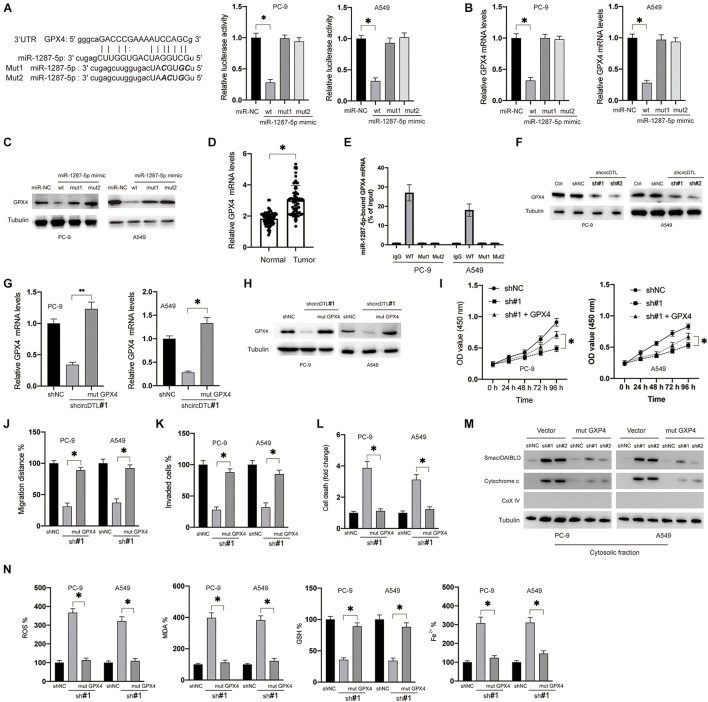
miR-1287-5p targets GPX4 to regulate both apoptosis and ferroptosis of NSCLC cells. **(A)** Predictive binding sites between miR-1287-5p and 3′-UTR of GPX4 (left), the interaction between miR-1287-5p and 3′-UTR of GPX4 was evaluated by dual-luciferase reporter assay (right). **(B)** NSCLC cells were transfected as indicated for 24 h, mRNA levels of GPX4 were measured by RT-PCR. **(C)** Protein levels of GPX4 were measured. **(D)** mRNA levels of GPX4 were measured in NSCLC and adjacent normal tissues. **(E)** RNA pull-down was conducted to evaluate the GPX4 mRNA that binds with miR-1287-5p. **(F)** NSCLC cells were transfected with shNC or shRNAs against circDTL (sh#1, sh#2), protein levels of GPX4 were measured. **(G)** mRNA levels of GPX4 were measured. **(H)** Protein levels of GPX4 were measured. **(I)** Cell viabilities were measured at different time points. **(J)** cell migration distance was calculated. **(K)** Several invaded cells were measured. **(L)** Cell death was measured. **(M)** the release of mitochondrial proteins was measured. **(N)** Levels of ROS, MDA, GSH, and Fe^2^ + were measured. Data were presented as mean ± SD. Experiments were performed at least three times. ^∗^*P* < 0.05; ^∗∗^*P* < 0.01.

In this study, it was attempted to identify the potential targets of miR-1287-5p. Various possible targets were predicted using bioinformatical techniques (TargetScan, miRDB), and GPX4 piqued the curiosity for this research for three reasons. Firstly, GPX4 was predicted in common by both analysis tools. Secondly, GPX4 was documented played a critical role in ferroptosis (Yang et al., 2014). Thirdly, GPX4 was also reported to be able to inhibit apoptosis *in vivo* (Ran et al., 2004). To verify whether miR-1287-5p can directly target GPX4, dual-luciferase reporter assays were conducted. Only the WT miR-1287-5p mimics, but not the mutants, inhibited GPX4 3′-UTR luciferase reporter activity (Figure 4A). Furthermore, transfection of the WT miR-1287-5p mimic into NSCLC cells inhibited the mRNA (Figure 4B) and protein levels (Figure 4C) of GPX4, whereas the mutants were ineffective (Figures 4B,C, right panel). RT-PCR results showed that mRNA levels of GPX4 in NSCLC tissues were much higher than adjacent normal tissues (Figure 4D). Moreover, GPX4 mRNA was shown to be concentrated in biotinylated WT miR-1287-5p but was unable to attach to mutant miR-1287-5p (Mut1, 2, 2, biotinylated) (Figure 4E). Similarly, it was observed that shRNAs against circDTL were also able to downregulate the GPX4 in NSCLC cells (Figure 4F). To investigate whether circDTL exerts its effects via the regulation of miR-1287-5p/GPX4 axis in NSCLC cells, a 3′UTR mutant GPX4 construct (mut GPX4) was transduced into shcircDTL stably expressing NSCLC cells. Mut GPX4 restored mRNA (Figure 4G) and protein (Figure 4H) expression of GPX4 in cells with knockdown of circDTL. According to cell viability assays, mutant GPX4 abolished knockdown of circDTL induced inhibition of cell viability (Figure 4I), migration (Figure 4J), and invasion (Figure 4K) in NSCLC cells. Meanwhile, increased cell death (Figure 4L) and release of mitochondrial proteins (Figure 4M) caused by the knockdown of circDTL were also abrogated by overexpression of mutant GPX4. Moreover, the effects of knockdown of circDTL on ROS, lipid peroxidation, GSH, and Fe^2+^ levels were also reversed by overexpression of mutant GPX4 (Figure 4N). These results suggested that circDTL exerts its effect via modulation of miR-1287-5p/GPX4 axis in NSCLC cells.

### Silencing of circDTL Increases Sensitivity to Chemotherapeutic/Ferroptosis Inducing Agents and Inhibits Tumor Growth *in vivo*

**FIGURE 5:**
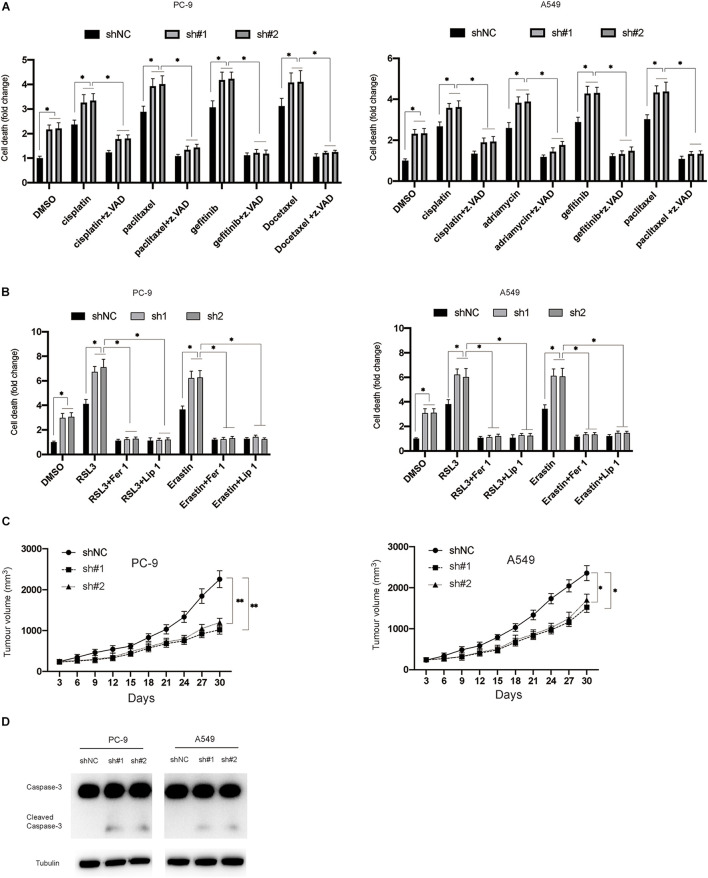
Silencing of circDTL increased the chemosensitivity of NSCLC cells and inhibited the growth of NSCLC *in vivo*. **(A)** NSCLC cells were exposed to different chemotherapy agents with or without z.VAD for 24 h and cellular death was measured. **(B)** NSCLC cells were exposed to RSL3 or Erastin with or without Fer-1/Lip-1 for 24 h, cellular deaths were measured. **(C)** NSCLC cells stably transfected with shNC or shcircDTL and inoculated into nude mice, tumor volumes were measured at different time points. **(D)** Xenografts were subjected to western blotting analysis. Data were presented as mean ± SD. Experiments were performed at least three times. ^∗^*P* < 0.05; ^∗∗^*P* < 0.01.

Furthermore, this study evaluated the effects of inhibition of circDTL on the chemosensitivity of NSCLC cells. In NSCLC cells, it was found that silencing circDTL enhanced cell death induced by different chemotherapeutic drugs (Cisplatin 10 M, Paclitaxel 10 M, Gefitinib 10 M, Docetaxel 10 M) (Figure 5A). Meanwhile, the cell death caused by those agents could be significantly inhibited by z.VAD (10 μM) (Figure 5A). Similarly, it was also observed that inhibition of circDTL increased sensitivity to ferroptosis inducers (Erastin 5 μM, RSL3 10 μM), and this effect could be blocked by ferroptosis inhibitors (Fer-1 10 μM, Lip-1 20 μM) (Figure 5B). The effects of downregulation of circDTL on the progression of NSCLC were then evaluated in nude mice xenograft models. The NSCLC cells transfected with shNC or shcircDTL were inoculated into nude mice, which revealed that shcircDTL markedly repressed the growth of tumors *in vivo*, as tumor sizes were significantly decreased when compared with the shNC group (Figure 5C). Furthermore, following silencing of circDTL, xenograft tumor tissues were exposed to western blotting, and it was shown that caspase-3 was activated (Figure 5D). These data indicated that downregulation of circDTL not only enhanced the chemosensitivity of NSCLC cells but also inhibited the growth of NSCLC cells *in vivo*.

## Discussion

Various studies have suggested that ncRNAs played essential roles in the initiation and progression of NSCLC (Anastasiadou et al., 2018). In this study, it was found that circDTL acted as an oncogene in NSCLC. It was found that knockdown of circDTL inhibited proliferation, migration, and invasion, and promoted both apoptosis and ferroptosis of NSCLC cells via modulation of miR-1287-5p/GPX4 axis.

Apoptosis is a programmed cell death that is triggered via two pathways namely the extrinsic and intrinsic pathways (Yu et al., 2016). The intrinsic pathway is strictly subjected to the regulation of Bcl-2 family proteins. In this study, it was found that silencing of circDTL leads to downregulation of Bcl-2, Mcl-1, and upregulation of Bax. These findings suggested that inhibition of circDTL induced apoptosis via the intrinsic pathway. Considering that most chemotherapeutic agents primarily induce apoptosis via the intrinsic route (Wu et al., 2021), it might explain why the silencing of circDTL increased the chemosensitivity of NSCLC cells. Ferroptosis is a novel identified form of cell death. Till now, there are only a few studies regarding the role of circRNAs in ferroptosis. For instance, Liu et al. found that circRNA cIARS regulated ferroptosis of liver carcinoma cells by interacting with the RNA-binding protein ALKBH5 (Liu et al., 2020). Zhang et al. (2020) showed that circTTBK2 regulates ferroptosis via binding with miR-761 and promoting ITGB8 expression in glioma cells. In this study, it was reported that silencing of circDTL promoted ferroptosis via regulation of miR-1287-5p/GPX4 axis in NSCLC cells.

Many studies have suggested that circRNAs exert regulatory effects through acting as competing endogenous RNAs (Memczak et al., 2013). Based on bioinformatics analysis, miR-1287-5p was predicted to bind with circDTL. Moreover, this study also found that circDTL was mainly located in the cytoplasm, and dual-luciferase reporter assays further confirmed the interaction between circDTL and miR-1287-5p. These findings suggested that miR-1287-5p acted as a tumor suppressor in NSCLC, which is similar to previous studies that had reported that miR-1287-5p inhibited the growth of breast and cervical cancer (Schwarzenbacher et al., 2019; Ji et al., 2020).

This study investigated the miR-1287-downstream 5p’s targets as well. The data suggested that GPX4 was a target of miR-1287-5p. Rescue experiments also confirmed that upregulation of GPX4 might restore the effects of downregulation of circDTL in NSCLC cells. GPX4 is an intracellular antioxidant enzyme that capable of reducing intracellular peroxidized phospholipids (Imai and Nakagawa, 2003). In recent years, many studies indicated that GPX4 plays an essential role in the process of ferroptosis. A study reported that inhibition of GPX4 led to ferroptosis especially in drug-resistant tumor cells (Viswanathan et al., 2017). Many agents, such as RSL3, ML162, and ML210, have been designed to target GPX4 to induce ferroptosis (Cao and Dixon, 2016). Overexpression of GPX4 reduced the release of smacSmac/DIABLO and cytochrome c, indicating that GPX4 influences intrinsic apoptosis. This finding is in line with a previous study that also reported that GPX4 blocked the release of cytochrome c from mitochondria (Nomura et al., 2000). In this case, these findings showed that GPX4 affects both apoptosis and ferroptosis. Another study revealed that high levels of GPX4 were correlated with the poor prognosis of NSCLC patients (Liu et al., 2018). We also found that GPX4 was significantly upregulated in NSCLC tissues than adjacent normal tissues. Hence, targeting GPX4 as a potential treatment for NSCLC might be a promising strategy.

## Conclusion

It can be concluded that this study is the first report that showed circDTL acted as an oncogene in NSCLC. Functional investigation showed that circDTL affected proliferation, migration, invasion, apoptosis, and ferroptosis of NSCLC cells. Mechanical examination showed that circDTL regulates both apoptosis and ferroptosis via miR-1287-5p/GPX4 axis. These findings have provided new insight into the development of cirRNA-based therapeutics against NSCLC.

## Data Availability Statement

The raw data supporting the conclusions of this article will be made available by the authors, without undue reservation.

## Ethics Statement

The studies involving human participants were reviewed and approved by the Ethics Committee of Ningbo University. The patients/participants provided their written informed consent to participate in this study. The animal study was reviewed and approved by The Animal Ethics Committee of Ningbo University.

## Author Contributions

WS conducted most of the experiments and draft the manuscript. MH repeated the experiments and verified the data. FJ conducted the statistical analysis. YY conducted the animal study. RY collected the clinical samples. YR designed and supervised this study. All authors contributed to the article and approved the submitted version.

## Conflict of Interest

The authors declare that the research was conducted in the absence of any commercial or financial relationships that could be construed as a potential conflict of interest.

## Publisher’s Note

All claims expressed in this article are solely those of the authors and do not necessarily represent those of their affiliated organizations, or those of the publisher, the editors and the reviewers. Any product that may be evaluated in this article, or claim that may be made by its manufacturer, is not guaranteed or endorsed by the publisher.

## Abbreviations

NSCLC: non-small cell lung cancer
circRNA: Circular RNA
miRNAs: MicroRNAs
ROS: reactive oxygen species
GPX4: Glutathione Peroxidase 4
MDA: Malondialdehyde
Fer-1: Ferrostatin-1
Lip-1: liproxstatin-1
RSL3: RAS-selective lethal 3
GSH: Glutathione.
